# Reports on Hospitals of the United Kingdom

**Published:** 1914-05-23

**Authors:** Henry Burdett


### REPORTS ON
### Hospitals of the United Kingdom.

By SIR HENRY BURDETT, K.C.B., K.C.V.O.

SERIES III.

### CROYDON GENERAL HOSPITAL.

This hospital was founded in 1867, the year previous to that in which we commenced our hospital work. It was rebuilt in 1904, and occupies a site which provides ample garden space in front of and behind the principal buildings. Architecturally it is one of the plainest hospitals; internally it is one of the brightest, airiest, and administratively encouraging hospital buildings in this country. Without waste of space there is yet ample room practically everywhere. Experience in inspecting teaches us that where possible the best plan is to ascend to the highest point of the buildings and then to go steadily through them floor by floor. The practical value of this plan can be driven home to the mind of anyone who will visit the Croydon General Hospital and pursue it.

### The Arrangement of Departments.

On the top floor the kitchens, nurses' and sisters' recreation rooms and much of the sleeping accommodation for the nurses and staff are provided. This arrangement of the departments makes a wonderful difference to the atmospheric conditions of a hospital. It should be followed in planning new and modern hospitals of the best type.

We had allowed none too much time for this particular inspection, but the atmosphere of the place is so wholesome and inspiriting, and the evidence of smart efficiency, which is the dominating note which permeates every department and portion of this hospital, was so apparent as to bring a freshness and inspiration by its encouragement to the inspector, who had, during many days, spent ten hours daily continuously in various hospitals. We visited every ward and the different sections of the hospital, including the casualty but not the out-patients' department, for we regard this latter department of hospital work as likely to disappear within a few years' time. It will, no doubt, be replaced by the establishment of a consultation branch, where cases of difficulty or special interest will be sent by practitioners for an opinion or a course of treatment, which can only be successfully pursued at a hospital.

So far as devoted and zealous work can make a hospital efficient and up to date the Croydon General Hospital is entitled to claim that its management is of the best. The wards are well kept, the furniture, though of the plainest, omits nothing which is essential or can be properly required for the comfort of the patients, and the gratifying condition of this hospital throughout is a matter which reflects the greatest credit upon the managers and officers and the nursing staff without exception. The cardinal principles which long years of experience have taught hospital workers, are to be found inculcated, taught, and practised throughout this institution. Take as an instance the management of the children's ward, which is unique in shape, probably octagonal or hexagonal, and presents a picture and teaches a lesson both in construction and in the management of children which might well be taken to heart by every hospital architect, and every matron, sister, and nurse who has the care of sick children, or is connected with the management of children's hospitals or children's wards. We have often said, and it is an absolute truth, that inspectors of children's hospitals, committees of management who control their affairs, as well as those within them, should note particularly whether the children are habitually quiet or whether they are frequently crying or wailing. A child never cries or wails unless it is in pain or discomfort, or wants food or attention of some kind. Hence a well-managed children's hospital can be, and ought to be, one of the most silent and cheerful of hospitals.

Our visit to the Croydon Hospital was paid soon after 10 A.M. on the morning of November 5, 1913, an hour which the Matron, Miss Mary Bird, who has grown up with the hospital, told us is, in her opinion, a time in the working day which most severely tests the quality of the management of any hospital of the kind. Be the quality of the test what you will, Croydon General Hospital responded to it in the most satisfactory manner, and, as we have said, everything was in order, and practically everything was as smart as a new pin. Not only the children but every patient in the hospital was contentedly happy, and exhibited evidence of their complete confidence that the very best was being done for each one of them, and that they were fortunate to be inmates of such an institution.

### A Great Example to Follow.

When we entered the hospital the first thing we noticed was a bust of an old fellow-worker and friend, Sir Thomas Eldridge. He was every inch of him a man; he hated inefficiency, and possessed the faculty not only of securing efficiency in connection with matters with which he was associated, but of teaching others by conviction to go and do likewise. We felt, as we left the hospital, that had Sir Thomas been with us during our inspection that morning—and may he not have been with us in spirit?—his heart would have rejoiced and been glad that he should have had the privilege of being one of the founders of the Croydon General Hospital, and has had the good fortune to be succeeded by committeemen and officers of all grades who are so whole-heartedly practising in their daily life, in the cause of this institution, the doctrines that he taught and the principle of each trying his or her best in all things as the secret of success.

### Equipment Details.

Of course there are some things which we could point out which might be changed, but they mainly affect equipment. For instance, the ward kitchens should have larger and a better type of sinks, and they might be smartened by block-tin coverings to the draining boards attached to them (and wherever else they may be in use throughout this hospital). We would further suggest that mackintosh sinks should be obtained, that it is not just to the high standard of efficiency attained in this hospital that the baths should have to be used in the absence of these necessary fittings. Hygienically the use of baths to wash mackintoshes is indefensible. We further consider that new and up-to-date slops and soil sinks of the best pattern should be gradually substituted for the old and somewhat inconvenient sinks now used for this class of work.

Having regard to the nature and character of the work that has to be done by the sisters in charge of the larger wards, we hope the committee will increase their rate of pay from a maximum of £35 to one of £45 per annum. We would venture further to recommend for the committee's careful consideration the reasonableness of the suggestion that the Matron should have the help of an assistant to be given the title and work, which in Miss Bird's opinion will tend to help her most effectually in maintaining the present high state of efficiency throughout the institution.

We were pleased to find that the accommodation provided for the resident medical staff and the matron is so good. It points to one underlying cause of the high efficiency of this hospital at the present time and to the spirit in which the upkeep has been gradually provided that the one defective and non-attractive bath is that used by the Matron and the resident medical staff. It is an old Rufford bath, we should say, which has done good service during decades of years. We imagine that Rufford's, if their attention were called to this bath, would gladly exchange it for a new one, for few better testimonials of the economical and wearing qualities of these baths could be found than in the history and service of the ancient one to which we refer.

### Finance.

We were sorry to notice that the accounts of this hospital are not kept upon the Uniform System, if we may judge from the printed statement in the report for the year ending June 30, 1912, which was given to us when we inspected the hospital on November 5, 1913. A hospital of the importance of Croydon ought to have its accounts kept on the Uniform System, so that the cost of maintenance, its revenue receipts, its working expenses, and the whole of its financial returns can be made comparable with other similar hospitals. Much is learnt in this way; many economies—and sometimes an increase of expenditure where that is justified and needed—result, with increased efficiency all round. It was our intention to have gone a little fully into the accounts of this hospital in order to show its present financial position. A reference to the accounts in the report showed they were not kept on the Uniform System, and compelled us to recognise that they at present leave much to desire from the point of view of those governors who take a businesslike and earnest interest in the working of this institution. Our fault with the accounts as they stand is that before comparing them with any other hospital an inquirer has to make his own analysis of each side of the accounts, to examine every item, and then to find from his want of knowledge of the details of particular items charged under particular heads that he cannot in fact make any precise comparisons at all. Under the Uniform System every item is posted to its proper heading, and the accounts show exactly what the ordinary income is, what the ordinary expenditure is, what each source of revenue for a voluntary hospital yields a year, what is the actual cost under the various heads of expenditure, the sub-heads of each heading being confined strictly to those items only which are given identically by each hospital where the accounts are kept upon this system. We certainly hope that before the report for the year ending June 30 next (why June by the way?) is published a committee will be entrusted with the duty of securing the introduction of the Uniform System and the publication of a statement of receipts and expenditure in the form this system secures. There are some of the wealthier and larger givers to hospitals who, as men of business, make a point of not contributing anything to a hospital nowadays which has not adopted the Uniform System.

